# The views of school leaders regarding gaining universal values in socio-cultural trips

**DOI:** 10.3389/fpsyg.2022.1033058

**Published:** 2022-09-28

**Authors:** Semattin Öztürk, Umut Akcil

**Affiliations:** Near East University, Faculty of Education, Nicosia, Cyprus

**Keywords:** intercultural, sensitivity, living values, education administrators, out-of-school activity

## Abstract

In the globalizing world, it is seen that life is more intertwined with different cultures. Therefore, intercultural sensitivity has vital importance for a peaceful harmonious common life. In order to ensure this sensitivity, societies must unite with common accepted values. In this regard, the adoption of universal values expressed by the UNESCO-UNICEF has particular importance. Therefore, schools have important responsibilities. Schools may prefer out-of-class activities in the development of positive sensitivity behaviors. In this study, the sensitivities developed by secondary-school students, who are the target group, toward living universal values after participating in out-of-school socio-cultural short-term trip activities were examined. The research adopts the phenomenological approach, which is one of the qualitative research methods. Interview techniques were used in accordance with this model. In line with the purpose of this research, the school administrators, who have the ability to witness the changes in the behavior of the children during and after the socio-cultural planned trip activities and who can best interpret them by filtering, have been reached. According to this criterion, 51 school administrators were selected. The results of the research revealed that short-term socio-cultural trips abroad have effects on living-universal values. It was observed that the positive behaviors in the school environment continued after the trip. We can say that, the development of sensitivity toward living-universal values also positively affects students’ intercultural sensitivity. As a result, it is recommended that school administrators give importance to such activities.

## Introduction

Rapid changes in information and communication technologies have made intercultural interactions compulsory in various fields, such as economy, politics, trade, and education. These rapid changes have necessitated the sustainability of universal peace and human life standards, thus increasing the importance of universal standards (Bayburt and Duman, 2020). It is considered important that societies with cultural differences should possess the skills, knowledge, and sensitivity necessary to live together. In this regard, education should be used for social life in societies to be successful (Rengi and Polat, 2014). Especially, Europe needs top-quality education and training systems to provide high quality skills that are in the line (Mirici, 2019).

Education plays an important role in transferring both universal and cultural values toward students (Koç, 2020). Another important task that schools have is to provide both national and universal values by running the in-class and out-of-class teaching process in the transfer of culture and value, and to prepare students for life within the framework of these values (Cihan, 2014). In this respect, it is seen that universal values that can be considered common factors that unite all societies, ensure intercultural communication, and provide value and respect for cultural differences are reflected in education systems. The most important of these values were determined by an international project supported by UNESCO prepared “Living Values Education Program (LVEP)” as part of the celebrations commemorating the 50th anniversary of the United Nations. Living values were prepared by the UNICEF Education Cluster and recognized by UNESCO; Raising awareness about basic values, such as peace, respect, love, happiness, freedom, honesty (trust), humility, tolerance, cooperation, responsibility, optimism, and unity is an important part of values education (Brahma Kumaris World Spiritual University, 1995). Besides the education process, the development of values is a part of the socialization process and is learned by observing (Joshi, 2007). For this reason, trying to give values education purely within the boundaries of the school may reduce the efficiency of the process (Koç and Budak, 2021). It is accepted that students learn more through the educational experiences conducted in out-of-school environments than those that occur in the traditional classroom (Cronin-Jones, 2000). Trip activities, which can also be used as an out-of-school learning environment, can achieve their purpose if they are planned effectively.

By embarking on a cross-cultural journey, it is possible to raise awareness or sensitize individuals toward the living common universal values. Trip activities for children teach important behaviors, such as recognizing events and people in their natural conditions, comprehending problems, critical thinking, approaching people and events with objective criteria, and gaining a sense of responsibility and cooperation (Behrendt and Franklin, 2014). Guest and Schneider (2003) stated that participating in extracurricular activities, both in and outside of school, generally, have positive behavioral consequences. It can be said that the activities applied in out-of-school (out-of-class) environments have many benefits both academically and in terms of gaining sensitivity toward universal values (Carrier, 2009; Bozkurt, 2017). Although programs that include a longer stay in a foreign country tend to positively affect students’ intercultural sensitivity, activities such as short-term trips and observations also enable students to gain first-hand experiences, which increases their motivation, strengthens their observation and perception sensitivity skills, and supports the development of social skills (Michie, 1998).

When previous studies are examined, studies on values education, teaching, awareness, and attitude development can be found. Values education is carried out with a formal education program. The contribution of planned out-of-school activities carried out within the framework of informal and hidden curriculum has been the subject of several studies. However, there are no studies showing how socio-cultural short trip activities affect students’ sensitivity to their values. This research is important in terms of both life practices and in order to eliminate this deficiency in the field.

### Universal values and intercultural sensitivity

“Values” are an existential source of power for our perception of the world and our drive to penetrate it. According to Höhler, it is possible to integrate ourselves into society with values (Höhler, 1994). The system of values, which forms the backbone of culture, provides unity and solidarity between people. Organizational psychology expert Hofstede (2001) defined culture as a set of common characteristics that affect the reactions of a group of people toward its environment and “culture in this sense includes values; systems of values are a core element of culture.” It is a social product and the result of interaction with people is the collection of learned behaviors. Thus, values can be learned and transmitted to the next generation (Ferraro, 2002). Values education, on the other hand, brings people who show good character examples, have moral values, and act with a sense of responsibility by emphasizing the universal values that all humanity has in common (Halstead, 1996).

Many institutions and organizations around the world have taken responsibility for values education and sensitization toward values and develop projects in this regard. UNESCO and UNICEF determined the living universal values as follows: justice and peace, respect, responsibility, love, truth and honesty, benevolence, and appreciation of cultural differences (Brahma Kumaris World Spiritual University, 1995; UNESCO, 2007).

Some researchers prefer to use the concept of intercultural sensitivity as a prerequisite skill (Hammer et al., 2003). Intercultural competence can be defined as the set of abilities needed to interact competently and effectively with people from other cultures (Wolff and Borzikowsky, 2018).

### The impact of cultural trip activities

Although attempts are made to teach the above-mentioned values in school with certain methods (Aspin, 2005), it is stated by theorists that the most effective method is learning by living with society. According to Feldman and Matjasko (2005), school/extracurricular activities direct students’ lives. They enable them to gain experience in terms of knowledge, attitude, and behavior, thus suggesting that these activities are an indispensable part of school. In addition to domestic cultural trip activities, studies have suggested that traveling abroad improves cultural sensitivity and related values (Anderson et al., 2006).

Trips are an important factor in understanding different cultures and recognizing their values. However, the harmonious coexistence of trip activities and culture and their planned planning may change behavior and attitude. Therefore, planned trips should be preferred (Kiper and Kiper, 2006).

In line with this information, in addition to the importance and contribution of planned socio-cultural trip activities to cultural differences and values. In the 2020–2021 academic year, this research was initiated due to the organized socio-cultural tour activities for thousands of student groups.

## Purpose of study

As a result of the evaluation of the gaps in the mentioned literature, the main purpose of this research is to determine the effects of planned out-of-school socio-cultural short trips on students’ experiences in terms of living universal values, according to the views of secondary school administrators. The research questions determined for this purpose are as follows;

1. What are the general opinions of the administrators about the different travel regions visited?
2. What is the level of sensitivity toward universal values developed by the students participating in the short-term socio-cultural trip activity?
3. What are the behaviors of the students during and after the short-term socio-cultural trip activities?

## Materials and methods

In this study, the opinions of school administrators were sought in order to understand the sensitivity indicators of students toward living universal values during and after socio-cultural trips. Therefore, this research was carried out with a phenomenological design, which is one of the qualitative research models. The purpose of qualitative research is to develop an understanding of how individuals make sense of their lives and to describe how they interpret their experiences (Denzin and Lincoln, 2018). The phenomenology design is a qualitative research approach that aims to highlight the perceptions and experiences of individuals according to their own perspectives. This process is usually carried out through interviews (Yıldırım and Şimşek, 2013).

### Participants

Participants, who are considered as data sources in phenomenological studies, are individuals or groups that experience the phenomenon on which the research focuses and can reflect this phenomenon outward (Manen, 1997; Ersoy, 2019). In line with the purpose of this research, the school administrators, who have the ability to witness the changes in the behavior of the children during and after the socio-cultural planned trip activities and who can best interpret them by filtering, have been reached. In this process, administrators can observe the changes in students before, during, and after the trip. In addition, administrators have more extensive school experience and can better recognize changes that occur in the school. According to this criterion, 51 school administrators were selected. These administrators worked in public schools in the north of Cyprus in the 2019–2020 and 2020–2021 academic years. The common feature of these administrators is that they participated in excursions with the students. For this reason, the criterion sampling method was used in the research. Since the opinions of the participants with common characteristics will be similar with criterion sampling, it will facilitate the interpretation of the phenomenon (Corbin and Strauss, 2007). It covered all types of schools at secondary level included in the trips, and therefore, the participants were drawn from a range of different levels: high school (*n* = 19), college (*n* = 16), and middle school (*n* = 16).

### Data tools

The interview technique was used in the data collection process. This technique involves compiling and making sense of people’s experiences, perceptions, thoughts, and expressions instead of testing an existing thesis (Büyüköztürk et al., 2012). The data collection tool consisted of two parts. In the first part, closed-ended questions were used to collect the demographic information of the participants, while the second part comprised open-ended interview questions. The created interview form was first distributed to a panel of five experts and their opinions were obtained. In order to determine the level of consensus, the percentage of agreement calculation suggested by Miles and Huberman (1994) was used. It was determined that the consensus among the experts was at the level of 90%. Afterward, a pilot study of 10 people was conducted. After the pilot study, the clarity and usefulness of the questions were tested. The final form was approved by the scientific ethics committee of the university. The questions used in the interview were as follows:

1. Were your visits sufficient to provide cultural gains to students?
2. Which universal value sensitivity do you think your students’ participation in socio-cultural trips develops? Please explain in detail.

b. What are the reflections of the specified values on the school environment? Can you give examples?

### Process

Creswell (2012) stated that in phenomenological studies, it is very important for all participants to have experience of the phenomenon being studied. For this reason, schools at the secondary level of formal education that participated in socio-cultural trips were firstly determined. Our study included schools participating in planned trips from Cyprus to Turkey. Therefore, the National Education Department was consulted to select the relevant schools. One of our researchers is currently responsible for the organization of the planned socio-cultural tour activities. Therefore, the process of selecting of schools participating in the excursions could be made easily. In the second stage, the selection process of the participants who will form the working group was carried out. The following criteria were used during the selection of the administrative participants from all the schools: (1) must have participated in cultural trips planned by the national education department; (2) should be still working as an administrator, and (3) should be a volunteer. According to these criteria, 51 participants who agreed to participate in the study by filling out the consent form were reached. Such trips have been conducted routinely for many years during the summer semester (except for the 2020 summer semester, when the COVID-19 pandemic started). It was found that the school administrators participating in this research had at least 2 years seniority. For this reason, during the period in which the administrators had served, they had had interactions with the students who participated in the excursions for a certain period of time. This situation is sufficient in terms of observing the behavioral changes in students and making judgments. Trip activities covered five out of seven geographical regions of Turkey (Marmara, Central Anatolia, Mediterranean, and Aegean) and lasted between 5 and 7 days.

Data collection was performed during the 2020–2021 spring academic year. Due to the ongoing COVID-19 pandemic, it was not possible to conduct face-to-face meetings; therefore, after obtaining the necessary permissions from the National Education Department, this process was carried out online.

### Data analysis

Qualitative research is a research method that focuses on words and expressions in the collection and analysis of data and how often they are repeated (Bryman, 2012). For this reason, content analysis or descriptive analysis techniques are generally used for interview and observation data. It is a common choice to use the descriptive analysis method in research with a well-drawn theoretical-conceptual framework. Descriptive analysis was used in this study. In this method, the researcher analyses the frequency with which words are used and matches the words with categories (Hsieh and Shannon, 2005). Descriptive analysis was applied to the data obtained from the open-ended questions. In line with the recommendations of Miles and Huberman (1994), our analysis process consisted of three stages. In the first stage, errors were eliminated and second stage the analysis process was applied in; the data were visualized, whereby the words were extracted and the frequency of repetition was revealed and tabulated. In the last stage, the conclusion was drawn and confirmed by direct quotations.

In this research, the main themes/categories were predetermined as follows within the framework of living universal values accepted by UNESCO (1992); peace, respect, love, happiness, freedom, honesty, helpfulness, tolerance, patience, responsibility, simplicity, and unity.

The following method was followed when giving direct quotations.

HS: High school, C: College, SS: Secondary School.

Example: “…………….” (HS1).

#### Reliability and validity studies

As in all qualitative research, validity and reliability are very important in phenomenology research (Bloomberg and Volpe, 2008). For the research to be credible, internal validity, external validity, reliability, and objectivity criteria must be met (Miles and Huberman, 1994). The following procedures were applied for these processes.

*Internal validity*: Long-term interaction with the data is provided. Confirmation of the data was obtained from the participants.
*External validity:* The transferability of the findings is ensured by direct quotations. Exhibitor information is given in detail.
*Reliability:* Two researchers conducted the analysis at different times and places. Research method was explained in detail
*Impartiality:* An unbiased expert compared the results and comments. The researcher explained his role in the process.

## Findings

In this study, the general opinions of the participants about the trip activities, which are the first sub-purpose of the research, were obtained. The answers from interview question 1 were examined in this respect and the findings are presented in Table 1.

**Table 1:** Opinions on the duration of socio-cultural trips.

	Duration	Secondary School (n)	High School (n)	College (n)	Total (n)
**Decision**	Sufficient	7	11	9	27
	Partially sufficient	9	5	5	19
	Insufficient	1	3	2	5

As seen in the table above, the majority of the administrators stated that the time allocated for planned trip activities (between 5 and 7 days) was sufficient (*n* = 27), followed by those who found it to be partially sufficient (*n* = 19). A smaller proportion stated that the allotted time was insufficient (*n* = 5). In line with this finding, the managers considered the short-term activities planned to visit a foreign country sufficient. This period, during which students and administrators can spend time away from their families, can be considered reasonable in terms of economic and psychological factors in terms of the management of the trip. For this reason, this finding does not provide an indication about the reflection of these trip phalli cities on the value judgments of the students. For this, the second and third sub-objectives should be examined.

For the second sub-purpose, which is considered important in terms of the subject of the research, students’ participation in cultural trips and their sensitivity toward universal values were examined. For this reason, the answers given for interview question 2 were analyzed. The findings are presented in Table 2.

**Table 2:** Universal values toward which sensitivity is developed in socio-cultural trips.

**Theme**	***f***	**Participants**
**Honesty**	19	SS1,SS2,SS10,SS13,SS14,SS15,HS1,HS2,HS3,HS4,HS8,HS13,HS14,C11,HS16C14,C13,C15, C16, and C1
**Responsibility**	21	SS8, HS16, SS5, SS7, C7, HS6, HS7, HS9, SS9, C8, HS10, C10, SS10, HS15, SS12, SS13, SS14, SS15, HS18, SS16, and HS19
**Patience**	36	C5, SS8, HS16, C1, SS1, SS2, C2, SS3, HS1, HS2, SS5, C3, HS3, C4, SS6, HS4, SS7, C7, SS8, HS8, SS9, C8, C10, HS12, HS13, HS14, SS10, SS11, SS13, SS14, HS16, C12, C13, SS15, HS18, and SS16
**Respect**	37	C5, SS8, HS16, SS6, C1, SS1, SS2, C2, SS3, HS1, HS2, SS5, C3, HS3, C4, SS6, HS4, SS7, C5, C7, SS8, HS8, SS9, C8, C10, HS12, HS13, HS14, SS10, SS11, SS13, SS14, HS16, C13, SS15, HS18, and SS16
**Helpfulness**	37	C5, SS8, SS7, C1, SS1, SS2, C2, SS3, HS1, HS2, SS5, C3, SS6, HS4, SS7, C7, HS6, SS8, HS7, HS8, HS9, SS9, C8, HS10, C10, HS11, HS13, HS14, SS10, HS15, SS12, SS13, SS14, C11, C13, SS15, HS18, and SS16
**Tolerance**	4	HS2, HS16, C15, and HS15
**Unity**	4	C5, SS8, HS19, and SS11
**Happiness**	5	C4, SS7, SS9, HS10, and HS11
**Self-Confidence**	4	HS18, SS2, SS7, and HS15
**Regularity**	3	HS6, C4, and C15
**Sharing**	7	HS2, HS9, SS3, SS9, SS16, C4, and C15
**Negative opinions**	5	HS5, HS17, C6, C9, and SS4

When Table 2 is examined, it is seen that 10 values have been identified in line with the opinions of the administrators. It was found that there was a unity of discourse about eight out of 12 values that reflected on the behaviors and sensitivities of the students after the short trips, in which UNESCO’s living universal values were taken into account. Values that were not mentioned as being reflected were “Peace, Freedom, and Simplicity.” In addition, “Self-Confidence (*f* = 4), Regularity (*f* = 3), and Sharing (*f* = 7)” values were reflected in the discourses. Several of the participants said that they could not see a positive contribution (*f* = 5). Some of the statements made by the participants regarding the values presented in Table 2 are as follows.

The remarkable answers we received about the value of responsibility were as follows:

*“They acted responsibly toward their friends and to themselves.”* (HS18).

*“On the way back from the trip, I saw that they behaved protectively and responsibly toward their friends within the boundaries of the school. In an unfamiliar environment, he developed responsibilities toward his friends.” (SS12).*

*“I observed that some of the students who took responsibility on the trips also took responsibility in the school environment, in the classroom environment and even outside the school.” (C7).*

The remarkable answers we received regarding the value of honesty were as follows:

*“I saw that the dialogue established at school with students who went on cultural trips is much more sincere. There were cordial conversations between them and their friends. For example, when an event was held, I saw that they were benevolent to each other.” (C10).*

“They are honest when communicating with their friends on trips. They answer the teacher’s questions honestly.” (HS15).

The remarkable answers we received regarding the unity value were as follows:

*“I think that the division of duties and the consciousness of acting together have developed. During the trip, the decision regarding the places to go in free time was made jointly. This behavior continued at school, and I saw those students acting together.” (SS8).*

*“According to my observations, when necessary, they share and do a job given by the teacher by acting together. They give importance to group work and are very enthusiastic. They are learning.” (C4).*

The remarkable answers we obtained regarding the value of trust and self-confidence were as follows:

*“Due to the trip, there were children who went abroad, perhaps for the first time, away from their families. These children developed their self-confidence. For example, when there is an activity in the school, these children take on a task with high self-confidence and say that they will do it.” (SS2).*

*“During the trip, I saw that protecting and helping each other improved mutual trust and responsibility.” (SS7).*

The remarkable answers we received with regard to the value of respect were as follows:

*“I noticed that students who experienced a different cultural environment behaved respectfully in every environment they entered, in museums and religious monuments. Even the most active student intrigued me with his solemn demeanor. I saw this behavior of respect in the community at their schools as well.” (HS15).*

*“I have seen them listen respectfully in the community and understand the beliefs and customs of the different cultures they encounter and behave respectfully. For example, they were silent when entering places of worship. When I got back to school, I saw that they were careful with their friends from different cultures.” (SS13).*

The remarkable answers we received about the value of patience were as follows:

*“The boy who patiently waits for his turn in a museum or similar places on trips. The child, who acts with respect without pushing his friends, has started to act with this awareness in the classroom. He was patient and respectful when entering and leaving the classroom” (C8).*

*“They are more patient and more respectful to their friends during the lesson, especially while listening to the lesson.” (HS15).*

*“I found that students were more patient with other schoolmates from Turkish culture in terms of listening to and understanding them.” (SS14).*

The remarkable answers we received about the value of tolerance were as follows:

*“In the trips made together, sometimes when someone was behind and couldn't catch up, they waited so as not to lose someone. I think it also shows that they are tolerant toward each other.” (C11).*

“*Before the visit, I saw that some of the students who had negative thoughts about cultures that contradicted their own were not nervous during the trip and were smiling.” (HS16).*

*“For example, they exhibited behaviors such as not disturbing during waking hours and travel hours, waiting for their turn to come, not disturbing their friends with whom they share the same room.” (H2).*

The remarkable answers we received about the value of benevolence were as follows:

*“The student, who did not want to help a friend who asked for help in the classroom before the trip, changed her attitude after the trip and became a helpful person. I can say that the trip contributed.” (HS15).*

*“I know that one of the students was sick on the trip. His friends were interested enough and tried to help. Because they didn't have families with them. Being in a different country strongly increases helping each other.” (C3).*

*“After returning from the trip, I found that students needed less direction to organize themselves in the activities and activities we did at school. They were helping each other with what needed to be done.” (C10)*

The remarkable answers we obtained about the sharing value were as follows:

*“I have witnessed positive reflections in this direction. The students shared with their friends about the new places and cultures they visited and recommended them to go to such places. There are similar positive reflections in terms of understanding different cultures.” (HS9).*

*“Because they acted collectively, they were cooperative toward each other in all matters. Such trips and programs develop these features. As a matter of fact, in school activities, they shared a given task among themselves” (SS16).*

*“Students on excursions learn to share best in these environments.” (C4).*

The remarkable answers we obtained regarding the value of happiness were as follows:

“*After these trips our children make, the positive changes at home are communicated to us by their families. While on the trip, some of our parents had concerns, but after returning home, they mostly shared their satisfaction with us. Both the child and the family were happy*.” (SS9).

“*There were positive reflections of the cooperation during the trip. Especially the students were happier and smiling*.” (SS7)

“*I see Çanakkale as a place that all young people should visit and the students I went to with my own school also returned from this trip incredibly happy and the warm and sincere behavior of the local people there made us very happy*.” (HS11).

The remarkable answers we obtained with regard to the regularity value were as follows:

*“Students gain behavioral discipline during excursions, which reflects positively on the school classroom environment. I saw that the students who disrupted the classroom order behaved more positively after the excursion and followed the rules.”* (C15).

*“I saw that the students who participated in these trips were more patient and organized depending on the group leader. When they returned, they behaved more regularly at school and in the classroom. To give an example, I saw that they came to school more regularly every morning*.*”* (C4).

However, a small number of administrators did not express positive opinions about the value of such short trips for students:

“*My answer is short and clear: positive, huge gains cannot be expected in such short-term trip programs*.” (SS4).

“*I do not believe that it is possible to gain some values permanently in a short period of 5 or 6 days, but it can be an experience for them to assume their own responsibilities*.” (C6).

“There is no eternal reflection. Some values are not gained in a short time.” (HS17).

For the third sub-objective of the study, a comparison of the behaviors, which is common during and after the short-term cultural trip activities of the students, was made in terms of values. For this purpose, the answers given to interview question 3 were analyzed. The findings are presented in Table 3.

**Table 3:** Comparison of affective behaviors observed during and after sightseeing activities.

**Theme**	**Seen during the tour**	**Reflected in the school environment**
**Honesty**	Show sincerity	Speak up
**Responsibility**	Taking responsibility for their behavior Acting responsibly	Do the assigned tasks on time Willingness to take on the task
**Patience**	Listen patiently Waiting for the turn of behavior	Waiting for speaking turn Waiting for the turn of behavior
**Respect**	Waiting one’s turn Talk calmly Respecting differences	Speaking respectfully in mutual communication Asking permission to speak in class
**Helpfulness**	Offer help Take action in problem solving	Doing business at events Offer help
**Tolerance**	Empathize To value Be interested in differences	Paying attention to feelings and thoughts To value Not discriminating between faith and culture
**Unity**	Cry in harmony with the group Watch over your friends	Cooperate Working with the group
**Happiness**	Enjoying their experience Sharing of feelings	Being smiling in the classroom/school environment Sharing happiness with friends Willingness to participate in social activities
**Self-Confidence**	Making new friendships Displaying protective behaviors	Being more active at school Improve friendship relations
**Regularity**	Obey the rules Attend on time	Move regularly Not be late for school
**Sharing**	Culture sharing Material shares	Sharing tasks in the group Be in a positive mood Good discourses for different cultures

As seen in Table 3, based on the discourses of the administrators, the behaviors at the affective level, which are common during and after the short-term cultural trip activities, were compared. It can be seen that some affective behaviors continued at school, albeit in a limited manner, both during and after the trip. The direct quotations given below Table 3 can be examined with respect to these findings.

## Decision, conclusion, and recommendations

The aim of this study is to determine the effects of out-of-school socio-cultural short trips on students’ sensitivity within the framework of living universal values, according to the views of secondary school administrators. According to this purpose, the focus was on whether there was a difference in the sensitivity of the students both during and after the trip. In this context, three research questions were asked to the school administrators and they were asked to evaluate their students. Based on the findings, it can be said that eight of the 12 universal values determined while designing the research are reflected in the value judgments of the students. Apart from these, three different values were also mentioned. These values were: honesty, responsibility, patience, respect, helpfulness, tolerance, unity, happiness, trust and self-confidence, orderliness, and sharing.

As a general result, it can be said that a short-term socio-cultural trip programs create a certain level of sensitivity and awareness. Intercultural sensitivity is revealed to a great extent when traveling and being exposed to other cultures. However, the values obtained in daily life are much more important (Segura-Robles and Parra-González, 2019). Shuman et al. (2016), found a positive relationship between international experiences and students’ intercultural competences in their study, which they stated provides evidence that it influences students’ future intercultural competence development. In general, it can be said that city and cultural trips make a positive contribution to eliminating the judgments that prevent recognition and understanding (Gergin, 2017).

Another result found in this research is that it can be said that short socio-cultural trips have created a slight behavioral change in students’ values. The change occurred mostly in affective behaviors and is considered important in terms of intercultural sensitivity. Kennedy (2014), in his study on teacher opinions, emphasized the positive achievements of students’ interaction with the environment during a trip. In terms of intercultural sensitivity, the interaction state is mainly concerned with the emotional domain.

Intercultural sensitivity requires the development of appropriate and effective behavior in identifying and evaluating cultural differences. The values of benevolence, respect, and patience that emerged with this study were intensely expressed. Such behaviors are said to be important in intercultural sensitivity. According to Sarwari and Abdul Wahab (2017), individuals should consider and respect all cultural differences in order to gain a good level of intercultural sensitivity. Aşçı and Akyavuz (2021) on the other hand, identified the importance of developing positive attitudes toward different cultures, ensuring that they do not find differences strange and show helpful behaviors toward them when necessary.

In an increasingly globalized society, it has become particularly important that students to learn to behave in harmony at school (Anderson et al., 2006). Even if the culture of each school is different, the most important acquisition that should be gained to students is universal values (Saripudin and Komalasari, 2015). When visiting for studying or short-term activities in other countries, students are able to improve their experience new cultures with people from different countries and territories. They can expand relationships in many countries and prepare for a future ahead (Vo, 2022).

Socio-cultural excursions take their place among out-of-school learning activities. It is seen that the awareness or education of values can occur in or out of school. Since values education is a part of life, it is important that the values that are planned to transfer to students in schools are not only given in lessons but also in social and cultural activities outside the classroom (Koç and Budak, 2021). It would not be an exaggeration to say that students’ mobility toward another country enriches their perspectives by enabling them to both understand themselves and the culture they encounter more deeply (Özışık, 2017).

## Limitations and future research directions

This study was conducted on the basis of opinions of school administrators. The administrators tried to explain the effects of socio-cultural short trips on the universal values of the students. We can say that socio-cultural trips, which are organized in a planned way, increase the awareness of children in terms of living universal values and increase their sensitivity to a certain extent. In addition, the administrators stated that the sensitivities developed for some universal values after the trips continued in the school environment as well. In this study, a broad review of the literature was conducted and the relationship and importance of living universal values while defining intercultural sensitivity was explained. The development of sensitivity toward living universal values also positively affects students’ intercultural sensitivity. It should not be forgotten that the relationship between living universal values and intercultural sensitivity is explained at the theoretical level, and it can be suggested that relational research on these two concepts be planned using scales.

In the study, only the opinions of the administrators were obtained. Therefore, seeking the opinions of teachers can expand the findings of this study in this context. The inclusion of all stakeholders of the education system is important for planning for the future.

## Data availability statement

The original contributions presented in the study are included in the article/supplementary material, further inquiries can be directed to the corresponding author.

## Ethics statement

The studies involving human participants were reviewed and approved by Ethical Committee Board of Near East University. The patients/participants provided their written informed consent to participate in this study.

## Author contributions

SÖ: conceptualization, methodology, investigation, resources, and writing/original draft preparation. UA: validation, writing/review and editing, visualization, supervision, and project administration. All authors contributed to the article and approved the submitted version.

## Conflict of interest

The authors declare that the research was conducted in the absence of any commercial or financial relationships that could be construed as a potential conflict of interest.

## Publisher’s note

All claims expressed in this article are solely those of the authors and do not necessarily represent those of their affiliated organizations, or those of the publisher, the editors and the reviewers. Any product that may be evaluated in this article, or claim that may be made by its manufacturer, is not guaranteed or endorsed by the publisher.
